# Regularized Latent Adaptive Framework for Unsupervised Industrial Anomaly Detection via Multi-Scale Generative–Discriminative Learning

**DOI:** 10.3390/s26134151

**Published:** 2026-07-01

**Authors:** Leqi Chi, Tao Ma, Yuhang Lang, Xinran Lv, Xingfan Li, Xiaoguang Li

**Affiliations:** 1 School of Electronic Information Engineering, Changchun University, Changchun 130000, China; 240402207@mails.ccu.edu.cn (L.C.); 17835209338@163.com (Y.L.); 240402203@mails.ccu.edu.cn (X.L.); 2 School of Economics and Management, Harbin University of Science and Technology, Harbin 150000, China; 2420710211@stu.hrbust.edu.cn; 3 School of Mechanical and Power Engineering, Harbin University of Science and Technology, Harbin 150000, China; lxxxr0705@163.com; 4 School of Electrical and Information Engineering, Changchun Guanghua University, Changchun 130000, China

**Keywords:** unsupervised learning, industrial anomaly detection, generative adversarial networks, attention mechanism, latent space distribution, feature fusion

## Abstract

Industrial visual inspection relies heavily on unsupervised anomaly detection due to the scarcity of annotated defect samples. However, existing methods struggle to balance global structural consistency and local defect sensitivity, leading to limited accuracy in practical scenarios. To address this challenge, we propose a unified generative–discriminative framework that combines regularized latent space encoding with multi-scale discriminator-guided supervision. Specifically, a dynamic compactness regularization strategy constrains latent representations of normal samples into a compact manifold to suppress anomaly reconstruction, while a multi-scale discriminator provides hierarchical perceptual constraints to enhance fine-grained anomaly localization across different spatial resolutions. Here we show that the proposed method achieves 98.6% image-level and 98.4% pixel-level AUROC on the MVTec AD benchmark, outperforming state-of-the-art approaches. This framework provides a stable and effective solution for real-world industrial quality inspection.

## 1. Introduction

Currently, automated surface defect detection with machine vision has become a crucial part of modern industrial quality control, replacing subjective and inefficient manual inspections in key manufacturing sectors. Its applications are extensive, covering areas from semiconductor fabrication and aerospace engineering to automotive manufacturing. However, most systems rely on observing many examples of defects that are precisely labeled. That is a problem [1]. Real defects are rare, varied, and unpredictable. Generating enough labeled data is impossible. This causes the systems to fail to identify new or unusual defects. This limitation necessitates a shift to alternative learning frameworks [2]. In recent years, deep learning-based anomaly detection methods have become popular in various industrial contexts [3]. Despite these advances, achieving robust and generalizable detection remains challenging, particularly when the variability and fine-grained nature of real defects must be captured under strict data constraints [4,5,6].

Historically, the direct application of deep learning to this domain has been significantly constrained by the scarcity of available anomaly samples in real-world industrial settings [7,8]. In stark contrast to large-scale datasets [9] such as ImageNet, which contains over 14 million images, surface defect detection is often confronted with the small sample challenge, where datasets may comprise merely a few dozen exemplars [10,11,12]. Numerous unsupervised domain adaptation strategies have been proposed to mitigate this issue by leveraging unlabeled target-domain data to improve generalization [13,14]. Yet, domain adaptation alone cannot fully address the intrinsic modeling gap: capturing the underlying manifold of normal imagery with sufficient fidelity to reliably reveal subtle deviations [15,16,17]. Motivated by this challenge, researchers explore a series of feature-level solutions that leverage the semantic richness of generative latent spaces to construct a precise and expressive representation of normalcy [18,19,20]. These explorations further promote the application of generative models in unsupervised anomaly detection.

Overall, unsupervised anomaly detection methods are mainly divided into two categories. Including methods based on reconstruction and feature embedding. While generative reconstruction models intuitively identify anomalies via pixel-level discrepancies, their core limitation is the well-known phenomenon of “anomaly reconstruction”—powerful generators often learn to reproduce anomalous regions faithfully, thereby severely diminishing detection sensitivity. Conversely, feature embedding-based methods offer superior local discriminability but heavily rely on fixed, task-agnostic external priors, which often suffer from domain gaps when applied to specific industrial textures. Therefore, existing methods still struggle to effectively reconcile the inherent contradiction between global structural consistency and local defect sensitivity. A critical challenge remains unresolved: how to organically integrate the global generative consistency of a task-specific reconstruction model with the local discriminative strength of a learned multi-scale feature representation, while simultaneously avoiding reliance on externally pretrained priors.

To address these fundamental challenges, this paper proposes a novel unsupervised anomaly detection framework that leverages StyleGAN2-ADA [21] as a generative backbone while introducing regularized latent representation learning and multi-scale discriminative supervision for industrial anomaly detection. The core motivation of the proposed framework is to reconcile the trade-off between global structural consistency and local defect sensitivity through a coordinated multi-component design. First, a coordinate attention module (CAM) is incorporated into the encoder to enhance spatial feature representation and preserve long-range structural dependencies. Based on these enriched representations, the proposed regularized latent space (RLS) constrains normal samples to form a latent manifold, thereby suppressing anomaly reconstruction and improving latent space separability. To further balance compactness and reconstruction fidelity, a PI-based adaptive controller dynamically adjusts the regularization strength during training. Second, to overcome the limited local sensitivity of conventional discriminators, the multi-scale discriminator (MSD) provides patch-level adversarial supervision at multiple spatial resolutions, enabling enhanced perception of subtle defects. During inference, the anomaly score is computed as the Mahalanobis distance between a test feature and the learned normal distribution for that scale and location. These multi-scale anomaly maps are then fused to produce a final, high-resolution localization result. By modeling the distribution of features learned specifically for the task of discriminating normal reconstructions, the proposed framework effectively bridges the gap between reconstruction fidelity and anomaly sensitivity without relying on external task-agnostic priors.

The main contributions of this paper can be summarized as follows:

1. We propose a new regularized latent space (RLS) encoding module that adds compactness constraints to the encoder generator mapping to balance reconstruction accuracy and anomaly detection capability during training.
2. We design a two-phase generative–discriminative architecture that intelligently repurposes a pretrained generative model into a multi-scale discriminator (MSD), enabling fine-grained, localized anomaly perception.
3. We introduce a probabilistic patch-distribution modeling strategy that fits multivariate Gaussian distributions to the task-specific, multi-scale adversarial features extracted by the discriminator, yielding precise and interpretable defect segmentations.
4. We propose a unified encoding–generation–discrimination anomaly detection paradigm that integrates representation learning, image reconstruction, and anomaly localization within a single framework. Extensive ablation studies validate the effectiveness of the proposed design.

The remainder of this paper is organized as follows: Section 2 provides a comprehensive review of the recent literature related to unsupervised industrial anomaly detection. Section 3 introduces the overall architecture of our proposed generative–discriminative framework and details its specific algorithmic implementations. Section 4 outlines the experimental procedures and presents a thorough analysis of both quantitative and qualitative results. Finally, Section 5 summarizes our main contributions and concludes the paper.

## 2. Related Work

### 2.1. Unsupervised Models for Anomaly Detection

Due to the inherent unpredictability and extreme scarcity of annotated defect samples in real-world manufacturing environments, unsupervised learning has emerged as the dominant paradigm for industrial surface anomaly detection. Over the past few years, extensive research has been dedicated to modeling the distribution of normal data to identify out-of-distribution patterns. Broadly, existing state-of-the-art approaches can be classified into two predominant trajectories based on their core mechanisms: reconstruction-based methods and feature embedding-based methods. In this section, we provide a brief review of the recent advancements and inherent limitations within these two main lines of research.

Reconstruction-based approaches fundamentally operate on the assumption that models trained exclusively on defect-free samples will fail to accurately reconstruct anomalous regions. Classic architectures in this trajectory, such as Variational Autoencoders (VAEs) [22] and GANomaly [23], learn a generative manifold of normal data and subsequently identify anomalies by computing pixel-level discrepancies between the input image and its reconstructed output. DRAEM [24], a typical representative of generative models, utilizes simulated pseudo-anomaly strategies to enhance sensitivity to subtle surface defects. However, its generalization ability is essentially limited by the diversity and authenticity of synthetic defects, which leads to fuzzy localization due to domain differences when encountering anomalies of different scales. Recent studies have shown that combining multi-scale architectures with GANs can effectively improve fine-grained anomaly perception, as demonstrated in multi-scale surface inpainting [25]. However, it is difficult to balance the mask size with local contextual information, and it lacks explicit constraints on the distribution of feature space. Although these methods are very intuitive and adept at capturing the global semantic consistency of target objects, they all have a key bottleneck called anomaly reconstruction. The powerful generalization capabilities of modern deep neural networks enable these generators to often learn an identity mapping that faithfully reproduces even the anomalous regions. Consequently, the expected pixel-level discrepancies are minimized, severely diminishing the model’s sensitivity to fine-grained, localized defects.

To address the limitations of pixel-level reconstruction, feature embedding-based methods have emerged as a dominant alternative. These techniques typically leverage deep neural networks pretrained on large-scale datasets to extract rich, patch-level feature representations. State-of-the-art frameworks then construct a normative distribution of these deep features using various statistical modeling techniques: PatchSVDD [26] employs hyperspheres, SPADE [27] utilizes memory banks, and PaDiM [28] models multivariate Gaussian distributions. Furthermore, advanced methods like DifferNet [29] apply normalizing flows for precise probability density estimation within the feature space [30], detecting anomalies as outliers based on feature distances. While embedding-based methods offer superior local discriminability, their primary drawback lies in a heavy reliance on fixed, external priors [31]. Because the pretrained backbones are not tailored to the specific textural and structural characteristics of industrial surfaces, a significant domain gap often emerges, limiting overall performance. Additionally, the patch-centric nature of these methods frequently neglects the holistic structural integrity of the target object. Recognizing this persistent dichotomy—where reconstruction methods capture global structure but miss local details, and embedding methods excel locally but lack global coherence and task-specific optimization—our work seeks to bridge these two paradigms. By combining the generative fidelity of a task-specific model with the robust discriminative power of a learned, multi-scale feature space, our framework achieves highly precise localization without depending on external pretraining [32,33].

### 2.2. Attention Mechanisms for Feature Enhancement

In unsupervised anomaly detection, extracting discriminative and robust feature representations is crucial for distinguishing subtle defects from complex background variations. To enhance feature expressiveness, attention mechanisms have been widely integrated into deep learning architectures, allowing models to dynamically focus on informative regions while suppressing irrelevant noise. Early approaches, such as squeeze-and-excitation (SE) networks or convolutional block attention module (CBAM) [34], primarily rely on channel-wise re-weighting or simplistic spatial pooling. However, these methods often compress feature maps excessively, leading to a loss of fine-grained spatial details that are critical for accurate defect localization. Conversely, while global self-attention modules or vision transformers (ViT) can effectively capture long-range dependencies, they introduce prohibitive computational costs. To address this dilemma, the coordinate attention module (CAM) [35] has emerged as a highly efficient alternative.

Unlike conventional channels or self-attention mechanisms, CAM introduces direction-aware encoding along both the horizontal and vertical axes, enabling the network to retain precise location information while maintaining low computational overhead. This is particularly beneficial for structured industrial defects such as elongated scratches and linear cracks, where spatial continuity is essential. Moreover, compared to global self-attention [36], it offers a superior trade-off between spatial sensitivity and efficiency, making it suitable for deployment without high computational cost. And coordinate attention provides an optimal balance between spatial information retention, computational complexity, and localization accuracy for this task. Additionally, the latent space regularization constraint promotes compactness within the encoded manifold, further enhancing mapping stability and anomaly separability.

In summary, although existing latent space regularization approaches have demonstrated effectiveness in improving representation quality, most rely on fixed regularization objectives that cannot adapt to the evolving latent distribution during training. Motivated by these limitations, we propose a regularized latent adaptive framework. By integrating latent space regularization, multi-scale discriminative learning, and embedding-based anomaly localization within a unified generative–discriminative paradigm, our method effectively addresses these challenges and provides a comprehensive solution for industrial inspection.

## 3. Method

In this paper, our framework is built upon StyleGAN2-ADA, a choice motivated by its unique architectural advantages that are highly synergistic with our proposed innovations. Beyond its state-of-the-art performance in few-shot learning, it features a highly disentangled latent space W+, which provides an ideal manifold for our regularized latent space encoding to effectively enforce compactness and separability. Furthermore, its hierarchical, multi-resolution synthesis process offers a natural structural foundation for our multi-scale discriminator to perform fine-grained, patch-level analysis at native spatial scales. For a detailed step-by-step description of the training algorithm and hyperparameter updates, we provide the pseudo-code of the algorithm in the Supplementary Materials.

**Figure 1:**
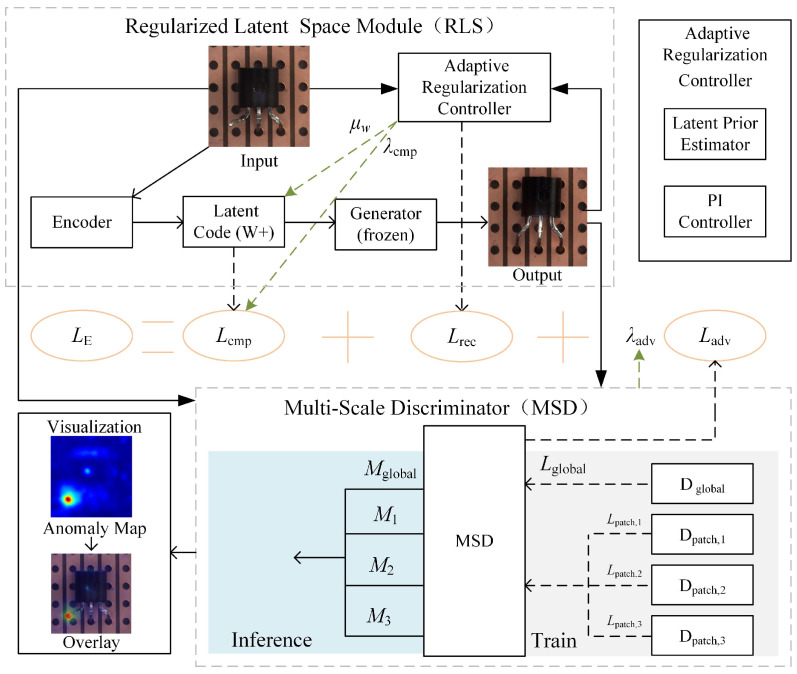
Overview of the proposed anomaly detection architecture. Solid arrows indicate the forward data flow; dashed black lines denote the computation of loss components; and dashed green lines represent the adaptive control signals for parameter updates.

This section describes the proposed unsupervised anomaly detection framework, which incorporates two main innovations: (1) a regularized latent space (RLS) encoding module, and (2) a multi-scale discriminator (MSD) that serves a dual role. During training, it provides adversarial supervision; during inference, its learned multi-scale features are utilized for localization via probabilistic distribution modeling. The overall anomaly detection architecture is illustrated in Figure 1.

### 3.1. The Architecture of Regularized Latent Space Encoding

**Figure 2:**
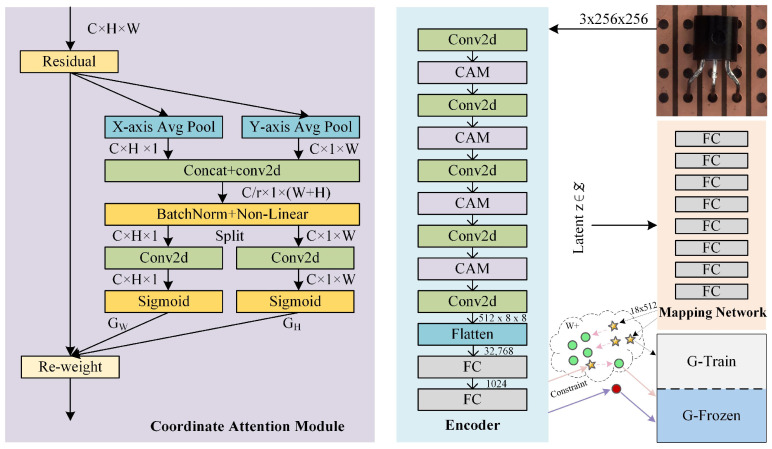
The network structure of the encoder for the latent space.

This section details the architecture and regularization strategies of the encoder, the overall structure of which is illustrated in Figure 2. The encoder projects an input image into the latent space W+. Let G denote the generator pretrained only on normal images, learning the manifold distribution $X_{\mathrm{normal}}\subset {\mathbb{R}}^{C\times H\times W}$. To obtain semantically meaningful and discriminative latent representations for anomaly detection, we propose an encoding design that integrates attention-driven feature extraction with explicit latent regularization. The encoder incorporates a coordinate attention module that embeds positional information into channel attention to capture long-range spatial dependencies, thereby enhancing the model’s ability to capture fine-grained spatial and contextual features. This design enables the encoder to capture long-range spatial dependencies and fine-grained contextual structures, thereby improving the separation between normal and abnormal latent manifolds. Additionally, the regularization constraint encourages compactness within the latent distribution, promoting stable mapping and enhanced anomaly separability.

In this section, let $w(x)=E(x)\in {\mathbb{R}}^{18\times 512}$ denote the latent code of an image in the latent space W+. For simplicity, these latent codes are represented as vectors in a high-dimensional space, and all operations such as addition and subtraction are performed element-wise.

To ensure that latent representations of normal samples remain compact and separable from anomalies, a compactness loss is imposed:

(1) $$L_{\mathrm{cmp}}\;=E_{x∼X_{\mathrm{normal}}}{\left\|E(x)-\mu_{w}\right\|}_{2}^{2}$$

where $\mu_{w}$ denotes the global centroid of the normal latent manifold. This centroid is dynamically estimated via an exponential moving average (EMA) over the mini-batches. At each training step *t*, given a mini-batch of normal samples $B_{t\;}$, the centroid is updated as follows:

(2) $$\mu_{w}(t)=(1-\alpha )\mu_{w}^{\left(t-1\right)}+\alpha E_{x\sim B_{t}}[w(x)]$$

where $E_{x\sim B_{t}}[w(x)]$ denotes the empirical mean of the latent codes within the mini-batch $B_{t}$, and α∈(0,1) is the momentum parameter.

A reconstruction constraint is also applied to maintain the generator fidelity:

(3) $$L_{\mathrm{rec}}=E_{x∼X_{\mathrm{normal}}}{\left\|x-\hat{x}\right\|}_{1}$$

where $\hat{x}$ is defined as $\hat{x}=G(E(x))$.

To dynamically balance reconstruction fidelity and latent compactness, we introduce an adaptive adjustment scheme for the compactness weight $\lambda_{\mathrm{cmp}}$. Inspired by control theory [37], we formulate this adjustment as a discrete proportional integral (PI) control problem. Specifically, the controller updates $\lambda_{\mathrm{cmp}}$ based on the deviation of the current reconstruction error, $L_{\mathrm{rec}}(t)$, from a predefined target threshold, τ.

The error term at training step t is defined as:

(4) $$e(t)=L_{\mathrm{rec}}(t)-\tau$$

To prevent the issue of integral wind-up—where the unbounded accumulation of past errors can lead to instability—we approximate the integral term using an EMA with a momentum parameter β∈(0,1):

(5) I(t)=β⋅I(t−1)+(1−β)⋅e(t)

Here, this acts as a leaky integrator, effectively maintaining a history of recent errors while gradually decaying the influence of older ones, ensuring bounded and stable control dynamics.

The final PI update rule for $\lambda_{\mathrm{cmp}}$ is then formulated as:

(6) $$\lambda_{\mathrm{cmp}}(t+1)=\lambda_{\mathrm{cmp}}(t)+\eta_{P}\cdot e(t)+\eta_{I}\cdot I(t)$$

where $\eta_{P}$ and $\eta_{I}$ are the proportional and integral gains. This dual-term feedback control ensures a smooth and stable adaptation $\lambda_{\mathrm{cmp}}$, mitigating oscillations and robustly guiding the training convergence.

The total regularization objective for the encoder, $L_{\mathrm{RLS}}$, is formulated as a dynamic weighted sum, where the PI-controlled $\lambda_{\mathrm{cmp}}(t)$ adaptively balances the two competing objectives:

(7) $$L_{\mathrm{RLS}}=L_{\mathrm{rec}}+\lambda_{\mathrm{cmp}}(t)L_{\mathrm{cmp}}$$

This formulation establishes reconstruction as the baseline objective and employs the weight $\lambda_{\mathrm{cmp}}(t)$ to dynamically regulate the intensity of the compactness constraint. This mechanism ensures a robust equilibrium, preventing the model from prioritizing reconstruction fidelity at the expense of latent separability, or vice versa.

### 3.2. The Architecture of Multi-Scale Discriminator

**Figure 3:**
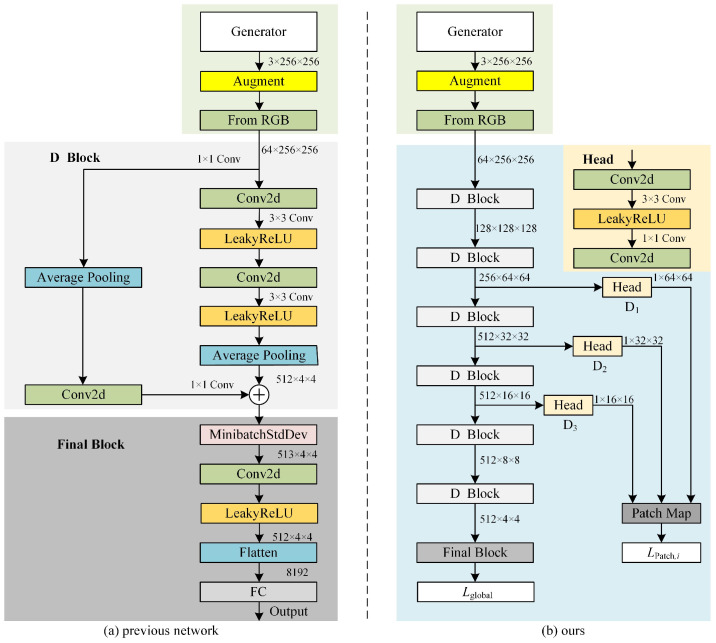
The network structure of the multi-scale discriminator.

To address the specific challenges of industrial anomaly detection, where anomalies can manifest as both large-scale structural deviations and subtle textural inconsistencies, a standard single-output discriminator is insufficient. We therefore propose a multi-scale discriminator, designed to enforce realism across a hierarchy of feature resolutions. Figure 3 illustrates the structure of the discriminator.

The discriminator is composed of two primary components: a deep residual backbone and a set of parallel projection heads. The backbone is constructed by stacking a series of StyleGAN2-based residual D Blocks. Figure 3, on the left, progressively downsamples the input image from 256 × 256 to 4 × 4, extracting increasingly abstract features at each stage. This design ensures powerful and stable feature representation. To achieve a better balance between global realism and local texture sensitivity, while the global branch evaluates overall image realism, the patch-level heads focus on fine-grained texture inconsistencies, thereby enhancing the model’s capability in local anomaly perception. Building upon this backbone, we introduce three projection heads, denoted as $D=\{D_{1},D_{2},D_{3}\}$. As illustrated in Figure 3, right, these heads branch off from the outputs of intermediate D Blocks, receiving features at resolutions of 64 × 64, 32 × 32, and 16 × 16, respectively. Each head, implemented as a simple 3 × 3 convolutional layer, projects the multi-channel feature map into a single-channel, patch-wise realism map. This forces the generator to produce plausible textures and local structures at multiple scales. Concurrently, the main branch of the backbone continues to the final block, which processes the most abstract 4 × 4 features to produce a single, global realism score, ensuring the overall coherence and semantic correctness of the generated image.

Following the principles of Geometric GAN [38] optimization, the Hinge loss is adopted to stabilize discriminator optimization and improve discrimination between real and reconstructed samples. The discriminator is optimized using the following objective:

(8) $$\ell_{\mathrm{hinge}}(D(x),t)=E[\max(0,1-tD(x))]$$

where D(x) denotes the discriminator output and t∈{+1,−1} indicates the label for real and generated samples, respectively. This formulation enforces a margin-based separation between real and synthetic data distributions, stabilizing the gradient dynamics during adversarial learning.

The total discriminator loss $L_{D}$ combines the contributions of the global path and the three patch-wise heads, with adjustable weighting coefficients $\lambda_{i}$:

(9) $$L_{D}=L_{\mathrm{global}}+\sum_{i=1}^{n}\lambda_{i}L_{\mathrm{patch},i}$$

$L_{\mathrm{global}}$ is the hinge loss applied to the discriminator’s global realism score. The first term penalizes the discriminator for assigning low scores to real images, while the second term penalizes high scores assigned to reconstructed images $\hat{x}$.

(10) $$L_{\mathrm{global}}=E_{x∼P_{\mathrm{data}}}[\max(0,1-D_{\mathrm{global}}(x))]+E_{\hat{x}∼P_{G}}[\max(0,1+D_{\mathrm{global}}(\hat{x}))]$$

For each scale, the discriminator outputs a spatial realism map $D_{i}$, and the loss is averaged across all pixel locations:

(11) $$L_{\mathrm{patch},i}={\mathrm{mean}}_{u,v}[\ell_{\mathrm{hinge}}(D_{i}{\left(x\right)}_{u,v}+1)+\ell_{\mathrm{hinge}}(D_{i}{\left(\hat{x}\right)}_{u,v}-1)]$$

This multi-scale hinge loss encourages each discriminative head to focus on its own receptive field, resulting in complementary supervision between global and local branches.

The adversarial component is formulated as:

(12) $$L_{\mathrm{adv}}=-E_{\hat{x}∼p_{G}}\left[D_{\mathrm{global}}(\hat{x})+\sum_{i=1}^{3}\lambda_{i}\cdot {\mathrm{mean}}_{u,v}D_{i}{\left(\hat{x}\right)}_{u,v}\right]$$

The encoder is optimized jointly with a fixed generator using the RLS reconstruction loss and an adversarial objective that compels them to deceive the MSD at all scales:

(13) $$L_{E}=L_{\mathrm{RLS}}+\lambda_{\mathrm{adv}}L_{\mathrm{adv}}$$

Among them, $\lambda_{\mathrm{adv}}\in (0,1)$, there is an artificially regulated balance coefficient between implicit regularization and adversarial training.

The overall optimization objectives of the proposed adversarial framework are summarized as follows:

(14) $$\underset{E}{\min}\;\underset{D}{\max}V(E,D)$$

where V(E,D) denotes the adversarial value function that measures the discrepancy between real samples and their reconstructed counterparts. This objective encourages the encoder to produce latent codes that lead to realistic reconstructions indistinguishable from normal samples, while the discriminator learns to identify subtle deviations, thus establishing a balanced adversarial dynamic. This joint formulation enables the model to maintain global generative stability while improving local discriminative sensitivity, providing robust and fine-grained anomaly localization under unsupervised conditions.

### 3.3. Modeling the Feature Distribution

To achieve high-resolution anomaly localization, we adopt a probabilistic feature modeling strategy inspired by PaDiM [28]. However, a crucial distinction of our work is that we do not rely on a generic, pretrained backbone. Instead, we leverage the powerful, task-specific feature representations learned by our adversarially trained multi-scale discriminator. During a preliminary step after training, we build a statistical model of the normal feature distribution. All normal images from the training set are passed through our trained Encoder and MSD. For each of the n discriminator branches, $D_{i}$, we extract the intermediate feature maps and compute the patch-wise mean and covariance matrix for the features at each spatial location. This results in a set of n multivariate Gaussian models, each corresponding to a specific scale of normalcy.

During inference, a test image x is fed through the same E-MSD pipeline. For each $D_{i}$, we extract its feature map and compute the Mahalanobis distance of each patch-wise feature vector with respect to the corresponding pre-computed Gaussian model for that location and scale. This process yields independent, scale-specific anomaly score maps:

(15) $$M_{i}{\left(x\right)}_{u,v}=\sqrt{{\left(f_{i}{\left(x\right)}_{u,v}-\mu_{i,u,v}\right)}^{T}\Sigma_{i,u,v}^{-1}(f_{i}{\left(x\right)}_{u,v}-\mu_{i,u,v})}$$

where $f_{i}{\left(x\right)}_{u,v}$ is the feature vector at location u,v from the branch $D_{i}$, and $\left(\mu_{i,u,v},\Sigma_{i,u,v}\right)$ are the learned statistics.

The scale-specific anomaly maps are upsampled to the input image resolution and fused via averaging to produce the final, high-resolution anomaly map. This strategy leverages the statistical modeling of discriminator features, rather than just relying on reconstruction error, yielding interpretable and granular anomaly localization. Because E–MSD is trained under a regularized latent constraint, the normal features become more compact, improving the Mahalanobis separation between normal and anomalous patches.

### 3.4. Evaluation Metrics

In this study, multiple quantitative metrics are employed to comprehensively evaluate the performance of the proposed framework in anomaly detection accuracy. For anomaly detection performance, we adopt the area under the receiver operating characteristic curve (AUROC) [39] at both the image- and pixel-level. The AUROC is a threshold-independent performance indicator that measures the trade-off between the true positive rate (TPR) and the false positive rate (FPR).

The TPR and FPR are defined as follows:

(16) $$\mathrm{TPR}=\frac{\mathrm{TP}}{\mathrm{TP}+\mathrm{FN}}$$

(17) $$\mathrm{FPR}=\frac{\mathrm{FP}}{\mathrm{FP}+\mathrm{TN}}$$

where TP, TN, FP, and FN denote the number of true positives, true negatives, false positives, and false negatives, respectively. The AUROC value is calculated by integrating the TPR over the FPR range:

(18) $$AUROC=\int_{0}^{1}T\mathrm{PR}(\mathrm{FPR})d(\mathrm{FPR})$$

In this study, the AUROC is applied at two different granularities.

Image-Level AUROC: This metric assesses the model’s capability to classify an entire image as either normal or anomalous. For each test image, a single anomaly score is assigned. It reflects the framework’s reliability in binary industrial quality grading.

Pixel-Level AUROC: This metric evaluates the precision of anomaly localization. Each pixel in the anomaly map is treated as an individual classification sample and compared against the corresponding pixel in the ground-truth mask. It indicates the model’s ability to accurately segment defective regions from the background, regardless of the anomaly’s shape or size.

## 4. Experiments

### 4.1. Experiment Details

#### 4.1.1. Datasets

**Figure 4:**
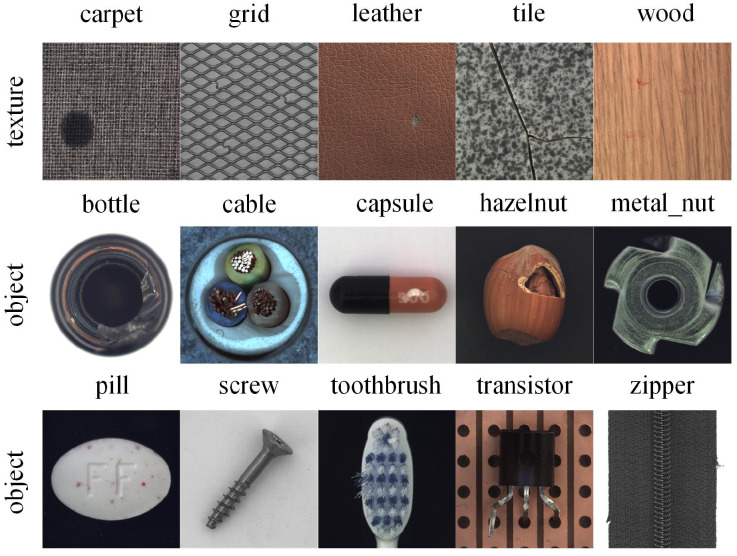
Representative images of the 15 categories in the MVTec AD dataset, including 10 object categories and five texture categories, which are used to evaluate the performance of the proposed unsupervised anomaly detection framework.

The MVTec AD dataset [40] serves as a comprehensive and standard benchmark in this field, containing 5354 high-resolution color images across 15 subcategories. Each subcategory consists exclusively of defect-free images for training, and test images containing various defect types. The dataset, as shown in Figure 4, spans a wide range of industrial products, providing a diverse set of challenges for evaluating the generative fidelity and reconstruction stability of models.

#### 4.1.2. Experimental Environment and Hyperparameter Settings

All experiments were conducted with an Intel^®^ Core™ i7 CPU and a single NVIDIA GeForce RTX 4090 GPU. The implementation was developed in Python 3.9.1 using PyTorch 2.1.0 and CUDA 12.1. All input images from the MVTec AD dataset were resized to a uniform resolution of 256 × 256 pixels.

Following the official configuration, our framework’s generator is built upon its architecture. This choice provides a powerful pretrained manifold of normal data and leverages its sophisticated synthesis mechanism, which utilizes weight demodulation instead of the original AdaIN to inject style information, thereby preventing normalization artifacts and ensuring high-fidelity image reconstruction. Unlike the original discriminator, R1 regularization is not applied to the global discriminator branch in our framework. Since the global discriminator is expected to learn anomaly-sensitive feature representations in addition to adversarial discrimination, excessive gradient smoothing may weaken its ability to capture subtle abnormal patterns. Therefore, the stability of the global branch is primarily maintained through latent space regularization and adaptive augmentation. However, R1 regularization is retained for the scale-specific discriminator heads to stabilize local adversarial learning.

Our training strategy involves a two-stage process. First, the generator is pretrained on anomaly-free images and then remains frozen to preserve the learned manifold of normal data. In the second stage, the pre-trained generator was frozen to preserve the learned normal image manifold, while the encoder and the multi-scale discriminator were jointly optimized. The AdamW optimizer was adopted with $\beta_{1}=0.0$, $\beta_{2}=0.99$, a learning rate of $1\times 10^{-4}$, and a weight decay of 0.01. Training was performed for 100 epochs with a batch size of 16. R1 regularization (γ=10) was applied to each scale-specific discriminator. All experiments were conducted with over 5 random seeds for reproducibility.

#### 4.1.3. Adaptive Discriminator Augmentation Strategy

We employ adaptive discriminator augmentation (ADA) strategically across the two training phases. During the generator pretraining stage, standard ADA is applied to enhance the generator’s robustness to photometric variations. However, for the subsequent encoder and optimization, we adopt a geometry-preserving augmentation policy. Specifically, destructive geometric transformations that alter the structural integrity of defects—such as large rotations, cropping, and cutout—are strictly disabled to ensure spatial consistency between training and inference. Instead, we utilize a constrained set of augmentations, including non-destructive geometric shifts and photometric perturbations. This tailored strategy prevents the model from learning invariance to critical defect geometries while maintaining sufficient regularization to stabilize adversarial training.

**Figure 5:**
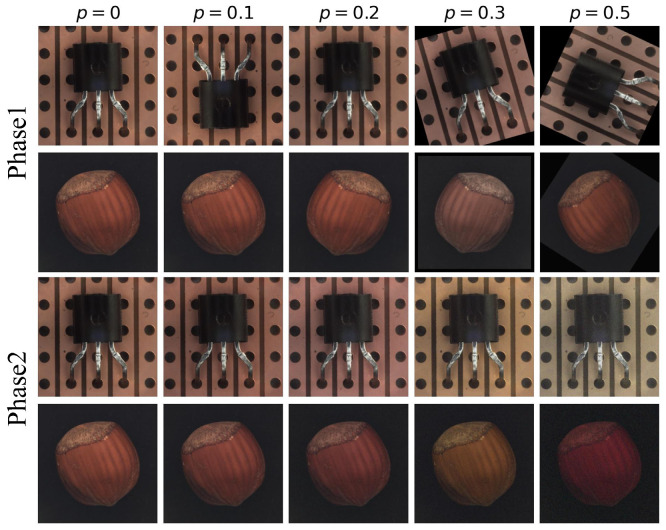
The visual effects of adaptive data augmentation on sample images.

Considering the task requirements of industrial anomaly detection, excessive enhancement is harmful to model training, and the strategy of the original ADA has been optimized. As shown in Figure 5, as P increases from 0 to 0.5, texture deformation and color perturbation become progressively stronger, introducing richer variations while maintaining the overall structural consistency of normal samples.

### 4.2. Anomaly Detection Results

**Figure 6:**
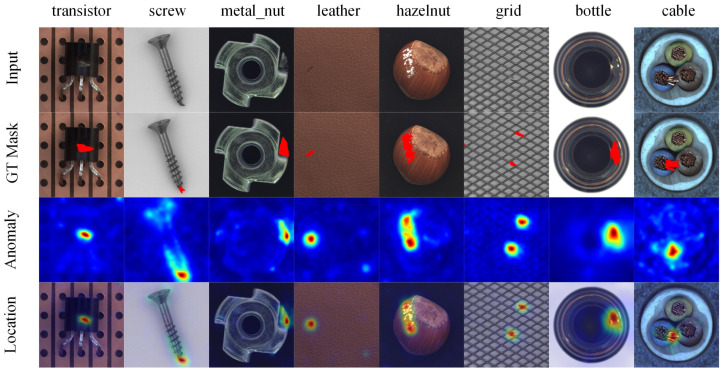
Qualitative anomaly localization results produced by the proposed method on representative samples from the MVTec AD dataset. From top to bottom, each group shows the input image, the ground-truth mask, the predicted anomaly map, and the corresponding localization result.

Figure 6 visualizes the anomaly localization results of representative objects in the MVTec AD benchmark. The figure illustrates the input images, ground-truth masks, predicted anomaly maps, and their overlaps, respectively. The visualizations demonstrate that our model produces highly interpretable heatmaps with a sharp contrast between normal and anomalous regions. Whether dealing with distinct structural defects on objects or subtle textural variations on surfaces, our model precisely localizes the anomalous regions at a fine-grained scale without triggering false positives in normal areas. More comprehensive visualization results are available in the Supplementary Materials.

### 4.3. Comparison with Existing Methods

To validate the effectiveness of the proposed detection framework, we conducted extensive quantitative experiments, comparing our method with several state-of-the-art unsupervised anomaly detection approaches, including vis.expl.VAE [22], GANomaly [23], PatchSVDD [26], SPADE [27], PaDiM [28], and DifferNet [29]. All methods were evaluated under the same experimental settings to ensure fairness and reproducibility. For the discriminator loss, all patch-head weights were uniformly set to 1.0 to ensure that each scale contributes equally to the adversarial objective without introducing a preferential bias.

**Figure 7:**
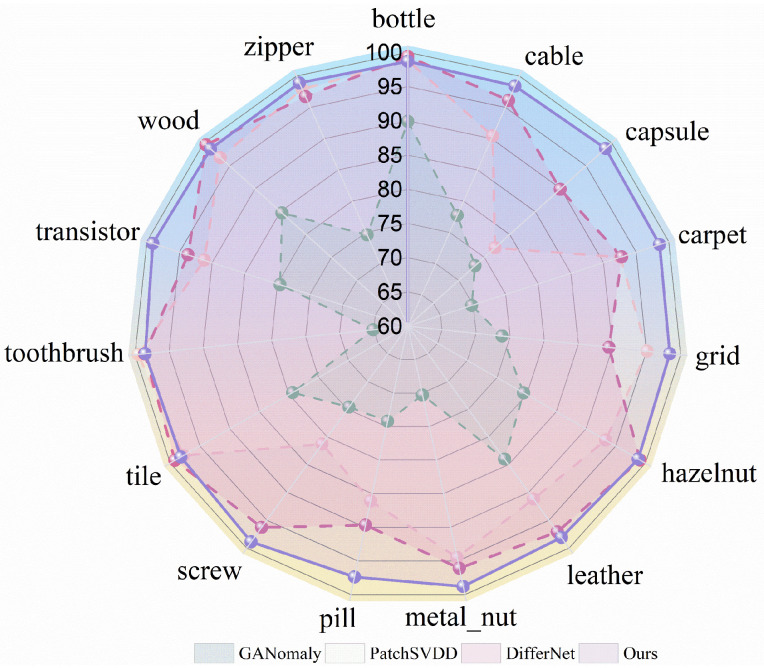
The radar chart provides a category-wise comparison of image-level AUROC performance, demonstrating the effectiveness in anomaly detection across different categories.

To quantitatively validate the effectiveness of the proposed framework, we compare its image-level anomaly detection performance against several state-of-the-art unsupervised baseline methods. As summarized in Table 1, our method establishes a significant performance with an average image-level AUROC of 98.6%, outperforming both reconstruction-based models and embedding-based approaches. This superior performance is further illustrated in Figure 7, where our method clearly forms the outermost bounding polygon, indicating not only peak accuracy but also remarkable stability across diverse categories.

**Figure 8:**
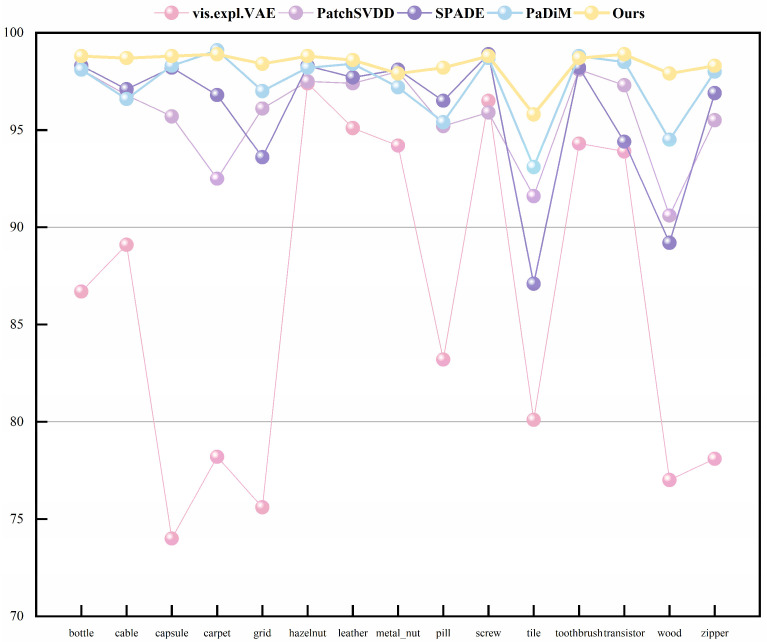
The line chart provides a category-wise comparison of pixel-level AUROC performance, demonstrating the effectiveness in anomaly localization across different categories.

To further evaluate the fine-grained defect localization capability of our framework, we compare the pixel-level AUROC scores against established baselines, as presented in Table 2. Our method achieves a state-of-the-art average score of 98.4%, outperforming strong feature-embedding methods such as PaDiM (97.3%) and SPADE (96.0%). Beyond the highest overall accuracy, the most striking advantage of our approach is its extraordinary robustness across diverse defect types, which is vividly illustrated by the line chart in Figure 8. As observed, the performance curve of our method consistently operates at the uppermost boundary with minimal fluctuation across all 15 categories. In stark contrast, competing baselines exhibit severe variance and sharp performance drops on challenging categories. This exceptional cross-category stability is a direct benefit of integrating our multi-scale discriminator with probabilistic patch distribution modeling. By extracting and statistically modeling multi-scale adversarial features, our framework effectively eliminates the reliance on generic pretrained backbones. It accurately captures localized textural inconsistencies and fine-grained spatial deviations, thereby yielding highly precise and robust pixel-level segmentations.

### 4.4. Ablation Study

#### 4.4.1. Effect of Adaptive Regularization Controller

To evaluate the efficacy of the adaptive regularization controller, we first establish the rationale for its key parameters: the proportional and integral gains ($\eta_{P}$, $\eta_{I}$), and the target threshold τ. These parameters were selected to ensure a critically damped response, preventing potential oscillations in the regularization weight $\lambda_{\mathrm{cmp}}$ while maintaining stable convergence. The selected configuration ($\eta_{P}=1\times 10^{-2}$, $\eta_{I}=1\times 10^{-3}$, τ=0.8 × initial mean error) consistently yielded stable training dynamics across different categories of the dataset. The τ serves as the operating setpoint of the controller, ensuring that latent compactness is progressively emphasized only after the reconstruction error approaches the desired target level, thereby maintaining a balanced trade-off between reconstruction fidelity and anomaly separability.

Unlike the fixed-weight and original proportional strategies, the dynamic PI controller adaptively adjusts $\lambda_{\mathrm{cmp}}$ to balance reconstruction fidelity and anomaly sensitivity. Table 3 compares the performance of different strategies for weighting the compactness loss $L_{\mathrm{cmp}}$.

For the best fixed-weight baseline, the compactness coefficient $\lambda_{\mathrm{cmp}}$ was set to 0.5, following the configuration [41,42], evaluated $\lambda_{\mathrm{cmp}}\in (0.1,1.0)$ on the transistor set. We observed that smaller values, such as 0.1, failed to effectively cluster normal samples, while larger values, such as 1.0, overly constrained the latent space, degrading reconstruction fidelity. Consistent with prior work that empirically selects a balanced mid-range weight, this setting imposes a sufficient penalty to ensure latent compactness without overwhelming the reconstruction objective, thus avoiding posterior collapse, and provides the best trade-off between accurate reconstruction and anomaly discriminability, and is thus adopted as our default fixed weight.

In terms of tuning cost, the fixed-weight strategy is the most expensive, requiring an extensive grid search over $\lambda_{\mathrm{cmp}}$ for each new dataset. In contrast, the PI-controller, while introducing three new hyperparameters $\left(\tau ,\eta_{P},\eta_{I}\right)$, can be effectively managed with a simple set of heuristics. Therefore, while its initial setup cost is moderate, it is significantly more practical and generalizable than the exhaustive search required by the fixed-weight baseline. The P-controller offers the lowest tuning cost, but at the expense of performance and stability. The results demonstrate that introducing the proposed PI control significantly improves both image- and pixel-level AUROC scores, achieving the most stable convergence throughout training.

#### 4.4.2. Effect of Multi-Scale Discriminator

The introduction of the multi-scale discriminator significantly improves fine-grained defect localization. By aggregating discriminative responses from small, medium, and large receptive fields, it enhances sensitivity to subtle texture changes while maintaining robustness to scale variations. Table 4 lists the results of different scale discriminators. In terms of average pixel-AUROC, our method only improves the score by 1.7% over the main discriminator.

Overall, the quantitative results verify that our method not only achieves state-of-the-art detection accuracy but also maintains stable training dynamics under limited normal data, which is essential for real-world industrial inspection scenarios.

## 5. Conclusions

In this work, we presented a regularized latent adaptive framework, a unified unsupervised anomaly detection framework that combines regularized latent encoding with multi-scale discriminative learning. By integrating compactness regularization into the latent space and enforcing detailed realism constraints through multi-scale discrimination, our proposed framework achieves a balanced synergy between generative reconstruction and discriminative localization. Extensive tests on the MVTec AD benchmark showed that our model not only outperforms existing GAN-based and embedding-based methods but also demonstrates excellent training stability and robustness under conditions with limited data. These results confirm that combining geometric regularization with adversarial hierarchy offers an effective approach for bridging the longstanding gap between reconstruction quality and anomaly sensitivity in industrial visual inspection. Beyond mere numerical improvements, the importance of our framework is in its conceptual contribution: it redefines unsupervised anomaly detection as a dual-space consistency challenge—where the latent manifold of normalcy must stay geometrically tight, while the perceptual space must maintain multi-scale realism.

While the proposed framework demonstrates significant efficacy, certain limitations warrant acknowledgment. First, the evaluation is primarily centered on the MVTec AD benchmark; thus, its generalization across a broader spectrum of industrial datasets and real-world scenarios requires further validation. Additionally, the current reliance on a regularized latent representation may constrain adaptability in the face of highly complex multi-instance distributions or non-stationary production environments. Furthermore, although the proposed framework achieves competitive detection performance, its computational efficiency and real-time inference capability have not yet been systematically investigated. Future work will therefore focus on optimizing the inference pipeline and conducting comprehensive FPS evaluations to facilitate deployment in practical industrial inspection systems. Nevertheless, this work successfully transcends the conventional reconstruction-versus-discrimination dichotomy. It suggests that the next frontier in anomaly detection lies not merely in increasing network depth or dataset scale, but in developing refined regularized representations that synergize generative and discriminative objectives within a unified optimization framework.

## Figures and Tables

**Table 1:** Anomaly detection performance (image-level AUROC [%]) on the MVTec AD dataset. “*” denotes a result with a backbone specifically selected for the task of image-level anomaly detection, which we could not reproduce. And the results for “Ours” are averaged over three independent runs, and the standard deviation reflects the variance across five random seeds.

Category	GANomaly	PatchSVDD	PaDiM	DifferNet	PaDiM *	Ours
bottle	89.9	98.8	-	**99.4**	-	98.7 _± 0.3_
cable	77.7	90.4	-	96.1	-	**98.4** _± 0.5_
capsule	73.2	77.1	-	89.9	-	**98.8** _± 0.4_
carpet	69.8	92.6	-	92.8	-	**98.6** _± 0.2_
grid	73.8	95.1	-	89.5	-	**98.4** _± 0.6_
hazelnut	79.5	93.3	-	**99.1**	-	98.8 _± 0.4_
leather	84.0	91.2	-	97.1	-	**98.1** _± 0.3_
metal nut	70.3	94.5	-	96.1	-	**98.8** _± 0.3_
pill	74.2	86.1	-	89.7	-	**97.4** _± 0.5_
screw	74.6	81.3	-	96.3	-	**98.9** _± 0.2_
tile	79.4	97.8	-	**99.2**	-	98.2 _± 0.4_
toothbrush	65.1	**99.7**	-	98.6	-	98.5 _± 0.3_
transistor	79.6	91.2	-	93.7	-	**99.1** _± 0.2_
wood	84.7	96.8	-	**99.6**	-	98.7 _± 0.4_
zipper	74.6	97.9	-	96.7	-	**98.9** _± 0.3_
average	76.7	92.3	95.2	95.6	97.9 *	**98.6 _±_** _0.4_

**Table 2:** Anomaly detection performance (pixel-level AUROC [%]) on the MVTec AD dataset.

Category	vis.expl.VAE	PatchSVDD	SPADE	PaDiM	Ours
bottle	86.7	98.1	98.3	98.1	**98.8** _± 0.3_
cable	89.1	96.8	97.1	96.6	**98.7** _± 0.4_
capsule	74.0	95.7	98.2	98.3	**98.8** _± 0.3_
carpet	78.2	92.5	96.8	**99.1**	98.9 _± 0.2_
grid	75.6	96.1	93.6	97.0	**98.4** _± 0.4_
hazelnut	97.4	97.5	98.3	98.2	**98.8** _± 0.3_
leather	95.1	97.4	97.7	98.4	**98.6** _± 0.4_
metal nut	94.2	98.0	**98.1**	97.2	97.9 _± 0.5_
pill	83.2	95.2	96.5	95.4	**98.2** _± 0.4_
screw	96.5	95.9	**98.9**	98.7	98.8 _± 0.3_
tile	80.1	91.6	87.1	93.1	**95.8** _± 0.5_
toothbrush	94.3	98.1	98.2	**98.8**	98.7 _± 0.3_
transistor	93.9	97.3	94.4	98.5	**98.9** _± 0.3_
wood	77.0	90.6	89.2	94.5	**97.9** _± 0.4_
zipper	78.1	95.5	96.9	98.0	**98.3** _± 0.4_
average	86.2	95.8	96.0	97.3	**98.4** _± 0.4_

**Table 3:** Comparison of the regularization controller parameter.

Controller Type	Configuration	Image-AUROC	Pixel-AUROC
No $L_{\mathrm{cmp}}$	$\lambda_{\mathrm{cmp}}=0$	95.1	93.9
Fixed Weight	$\lambda_{\mathrm{cmp}}=0.5$	97.0	96.2
P-Controller	$\lambda_{\mathrm{cmp}}(t)$	98.1	97.5
Ours	$\lambda_{\mathrm{cmp}}(t)$	**98.6**	**98.4**

**Table 4:** Comparison of pixel-AUROC for different scale discriminators in the MVTec AD transistor data.

Model Configuration				Avg Pixel-AUROC	Bent Only	Cut Only	Damaged Case Only	Good Only	Misplace Only
*D*_global_	*D*_1_	*D*_2_	*D*_3_						
√	-	-	-	97.2	96.8	96.4	97.2	98.3	97.4
-	√	√	√	97.8	98.0	97.6	97.9	98.6	97.1
√	√	-	-	98.1	98.1	98.2	97.6	98.7	97.8
√	-	√	-	98.6	98.9	98.5	98.2	99.0	98.4
√	-	-	√	98.0	98.3	97.8	97.4	98.8	97.9
√	√	√	√	98.9	98.5	98.8	98.5	100	98.7

## Data Availability

Publicly available datasets were analyzed in this study. The MVTec dataset is available at https://www.mvtec.com/research-teaching/datasets/mvtec-ad. (accessed on 23 June 2026). Additional processed data supporting the findings of this study are available from the corresponding author upon reasonable request.

## Supplementary Materials

The following supporting information can be downloaded at: https://www.mdpi.com/article/10.3390/s26134151/s1. Figure S1. The visual latent distributions in MVTec AD transistor data. Figure S2. Explanatory analysis and curve graphs of four categories in the MVTec AD dataset. Figure S3. Visual display of a histogram in the MVTec AD transistor data. Figure S4. The proposed method produces qualitative anomaly localization results on supplementary samples in the MVTec AD dataset. From top to bottom, each group displays the input image, ground truth mask, predicted anomaly map, and corresponding localization results. Algorithm S1. Training procedure. Algorithm S2. Inference and anomaly localization procedure. Table S1. Ablation study on different augmentation strategies during the joint training phase. Performance is reported as pixel-AUROC (%) on the MVTec AD dataset with 5 random seeds for reproducibility. Table S2. Evaluation of reconstruction fidelity on normal samples with 5 random seeds for reproducibility. References [42,43,44,45,46] are cited in the supplementary materials.

## Abbreviations

The following abbreviations are used in this manuscript:

RLS	Regularized latent space
MSD	Multi-scale discriminator
CAM	Coordinate attention module
ADA	Adaptive discriminator augmentation
AUROC	Area under the receiver operating characteristic curve
