# Supplementary material for: Regularized Latent Adaptive Framework for Unsupervised Industrial Anomaly Detection via Multi-Scale Generative–Discriminative Learning
Source: Sensors (Basel). 2026 Jul 1;26(13):4151. doi: 10.3390/s26134151 (PMC13364467; doi:10.3390/s26134151)
Supplement: Supplementary file 1 [file sensors-26-04151-s001.zip › sensors-4372475-supplementary.pdf]

Supplementary Material

1.1. *Interpretability Analysis*

To visually validate the effectiveness of RLS encoding, we employ t-SNE to project the high-dimensional latent codes of the transistor test set into a 2D space.

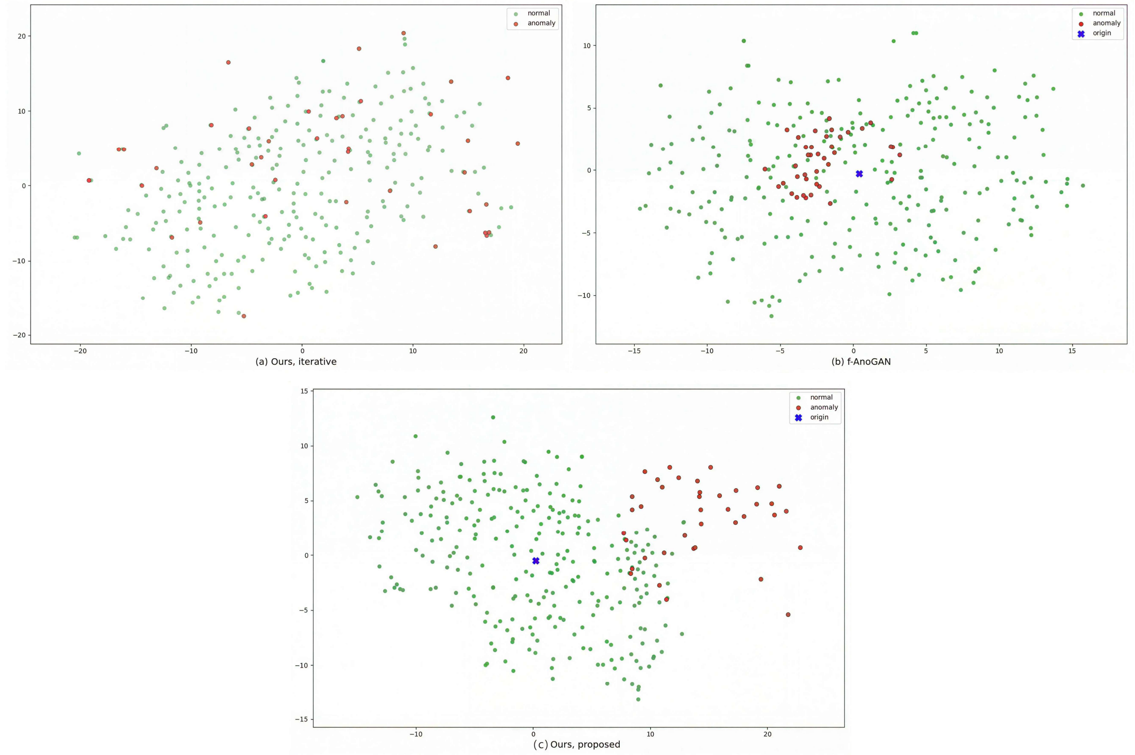

Figure S1. The visual latent distributions in MVTec AD transistor data.

(a) For a baseline model trained without our compactness constraint L_{cmp} , the latent representations of normal (green) and anomalous (red) samples are inextricably mixed, demonstrating a lack of inherent separability.

(b) With a weakly regularized model, f-AnoGAN [1], we observe an initial emergence of structure, where anomalous samples begin to form a loose cluster but remain heavily overlapped with the normal distribution.

(c) In stark contrast, our fully trained proposed method enforces a powerful compactness constraint, compelling all normal samples into a single, tight, and well-defined manifold. Crucially, this process simultaneously pushes all anomalous samples, regardless of their specific defect type, into a distinct and clearly separated region of the latent space. This visualization provides compelling qualitative evidence that our regularized latent space module successfully learns a discriminative latent space where anomalies are geometrically isolated from the learned distribution of normalcy.

Fig. S1. illustrates a comparative analysis of the latent distributions under three distinct configurations. As shown, the proposed regularized latent space encoding module enables the encoder to learn a more compact and stable latent representation that effectively preserves the structural characteristics of normal samples while amplifying deviations caused by anomalies.

We illustrate more categories of AUROC curve and visual latent distributions in the MVTec AD.

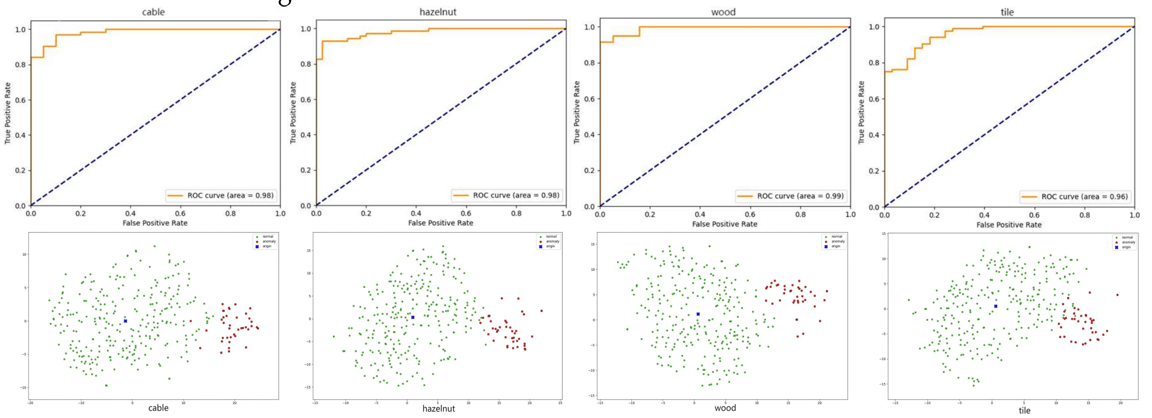

Figure S2. Explanatory analysis and curve graphs of four categories in the MVTec AD dataset.

We visualize the histogram representation of the complex data in Table 4 of the main text.

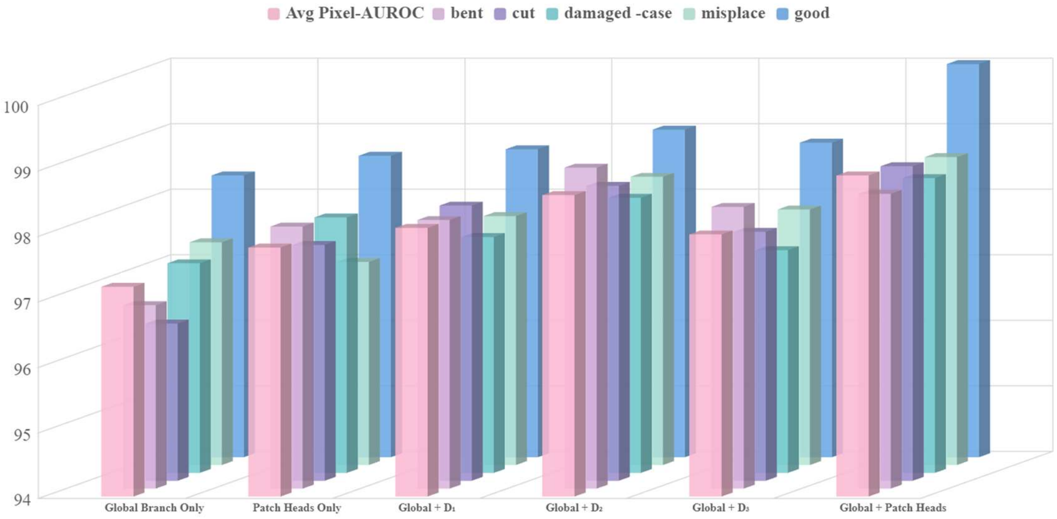

Figure S3. Visual display of a histogram in the MVTec AD transistor data.

The above Fig. S3. presents a detailed ablation analysis of the multi-scale discriminator (MSD) components across various defect subcategories of the MVTec AD transistor dataset. The results demonstrate that the full configuration, Global + Patch Heads, consistently achieves the superior performance, reaching a peak average Pixel-AUROC of 98.9%. A comparative analysis reveals that while the Global Branch Only provides a baseline for structural consistency, it lacks the sensitivity required for subtle textural anomalies like bent or cut defects. Conversely, the integration of individual patch heads significantly boosts localization precision at different spatial resolutions. Notably, the Global + D_2 configuration shows a marked improvement in detecting bent and cut categories, suggesting that intermediate receptive fields are particularly effective for mid-sized geometric deformations. The synergy between the global realism score and multi-scale patch-level supervision ensures that the model maintains high discriminative precision across diverse anomaly types, validating the necessity of our hierarchical adversarial framework for robust industrial inspection.

We show more categories of defect localization and heat maps in the MVTec AD dataset.

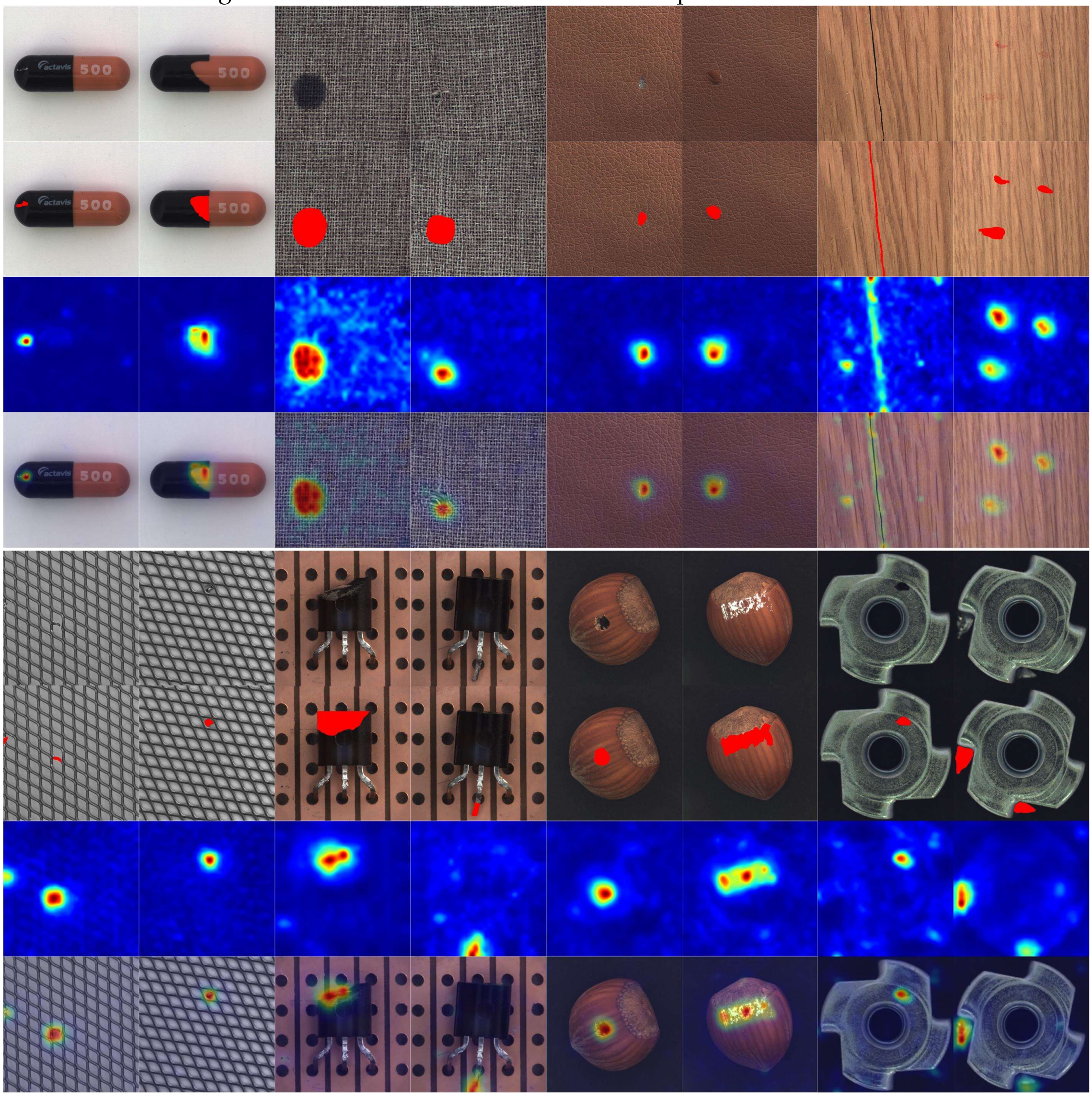

Figure S4. The proposed method produces qualitative anomaly localization results on supplementary samples in the MVTec AD dataset. From top to bottom, each group displays the input image, ground truth mask, predicted anomaly map, and corresponding localization results.

Algorithm S1 Training procedure

Train process

Inputs:

- Normal training set D_{normal}
- Mapping network f and generator G
- Encoder E
- Multi-Scale Discriminator $MSD = \{D_{\text{global}}, D_1, D_2, D_3\}$
- ADA policies $P_{\text{pretrain}}, P_{\text{phase2}}$
- PI-controller hyperparameters η_{P} , η_{I} , α , λ_{adv}
- Training hyperparameters: epochs, learning rates, batch size, etc.

Phase 1 — Pretraining the Generator on Normal Images

1. Initialize the mapping network f , generator G , and base discriminator D .
2. For epoch = 1 to E_{pre} :

    For each batch $X_{\text{real}} \sim D_{\text{normal}}$:

    (a) Apply ADA:

    $x_{\text{aug}} = ADA\_apply(x, P_{\text{pretrain}})$

    (b) Sample latent code $z \sim N(0,1)$, compute $w = f(z)$ and $x_{\text{fake}} = G(w)$

    (c) Update discriminator using hinge loss:

    $L_{\text{D}} \leftarrow HingeLoss\_D(x_{\text{aug}}, x_{\text{fake}}) + R1\_penalty$

    (d) Update generator:

    $L_{\text{G}} \leftarrow HingeLoss\_G(x_{\text{fake}})$

    end

    end
3. Freeze the pretrained generator G

Phase 2 — Joint Training of Encoder and MSD

4. Initialize encoder E , MSD, running centroid $\mu_w(t)$, and PI controller memory.
5. For epoch = 1 to N_{epochs} :

    For each batch x_{real} in D_{normal} :

    (a) Weak ADA:

    If enabled: $x_{\text{aug}} = ADA\_apply(x, P_{\text{pretrain}})$

    Else: $x_{\text{in}} = x_{\text{real}}$

    (b) Encode and reconstruct:

    $w = E(x_{\text{in}})$, $\hat{x} = G(w)$

    (c) Extract features from MSD:

    Real_feat = MSD(x_{in}), Fake_feat = MSD($\hat{x}$)

    (d) Update MSD using multi-head hinge loss:

    $L_{\text{D}} \leftarrow HingLoss(MSD(x_{\text{in}}), MSD(\hat{x}.detach()))$

    (e) Update MSD parameters using ∇L_{D}

    (f) Compute reconstruction loss:

    $L_{\text{rec}} \leftarrow \|x_{\text{in}} - \hat{x}\|_1$

    (h) Compute encoder adversarial loss:

    $L_{\text{adv}} \leftarrow -(D_{\text{global}}(\hat{x}) + \sum D_{\text{patch},i}(\hat{x}))$

    (i) Compactness loss:

    $L_{\text{cmp}} \leftarrow \|w - \mu_w\|^2$

    (j) Update λ_{cmp} via PI control:

    (k) Total encoder loss:

    $L_{\text{E}} \leftarrow L_{\text{rec}} + \lambda_{\text{cmp}} \cdot L_{\text{cmp}} + \lambda_{\text{adv}} \cdot L_{\text{adv}}$

    (l) Update E parameters using ∇L_{E}

    (m) Update latent centroid:

    $\mu_w \leftarrow (1-a)\mu_w + a \cdot \text{mean}(w.\text{detach}())$

    end

    end

Algorithm S2 Inference and anomaly localization procedure

Inference process

Inference

6. Encode input:

   $w = E(x)$

7. Reconstruct:

   If enabled: $\hat{x} = G(w)$

8. Extract multi-scale feature maps from MSD:

   $feat_t = D_i(x)$

9. For each spatial location (u,v) of each head i :

   Compute difference vector:

   $\delta = feat_i(u,v) - u_i(u,v)$

   Regularize covariance:

   $\sum^* = \sum_i (u,v) + \epsilon I$

   Mahalanobis score:

   $M_i(x)_{u,v}$

10. Upsample each $M_i(x)$ to the input resolution, normalize individually.

11. Fuse multi-scale maps (average):

    $M_{\text{feat}}(x)$

12. If reconstruction map enabled:

    $M_{\text{rec}}(x) = |x - \hat{x}|$, normalize

    Final score: $M(x) = aM_{\text{feat}} + (1-a)M_{\text{rec}}$ (Additional comparative test)

    Else:

    Final score: $M(x) = M_{\text{feat}}$

13. Image-level score:

14. Return image-level score and pixel-level anomaly heatmap.

1.2. Supplementary Experiments on Augmentation Strategy

To validate our design choice of disabling geometric augmentations during the E/MSD training phase, we conducted an ablation study comparing different augmentation policies. As shown in Table S1, applying the full suite of ADA transformations, particularly geometric ones, leads to a notable degradation in performance, especially on categories with structural anomalies.

Table S1 Ablation study on different augmentation strategies during the joint training phase. Performance is reported as Pixel-AUROC (%) on the MVTec AD dataset with 5 random seeds for reproducibility.

Augmentation Strategy	Description	Avg AUROC	cable (Structural)	carpet (Textural)
No Augmentation	None	$96.5_{\pm0.5}$	$96.4_{\pm0.3}$	$96.8_{\pm0.3}$
Full ADA	Geom + Color + Noise + Cutout	$95.8_{\pm0.8}$	$93.5_{\pm0.9}$	$97.0_{\pm0.7}$
Geometric Small	Rotation ±5°, Translation ±2%, Scale ±2%	$96.8_{\pm0.6}$	$95.0_{\pm0.8}$	$97.7_{\pm0.4}$
Geometric Large	Rotation ±30°, Translation ±10%, Scale ±10%	$94.0_{\pm0.8}$	$89.8_{\pm1.2}$	$95.0_{\pm0.6}$
Cutout only	Random masks applied with a size of 5–20% of the image area	$97.0_{\pm0.4}$	$95.5_{\pm0.7}$	$97.5_{\pm0.4}$
Ours	Brightness, Saturation, Weak Noise	**98.4**$_{\pm0.4}$	**98.7**$_{\pm0.4}$	**98.9**$_{\pm\mathbf{0.2}}$

We attribute this performance drop to the misalignment introduced by geometric transformations. Since our anomaly localization relies on precise spatial correspondence between the input image and the reconstructed/feature-mapped representations, large rotations or translations disrupt this pixel-wise alignment, confusing the MSD and the encoder. In contrast, our proposed Photometric Only strategy effectively improves robustness to lighting variations without compromising spatial consistency, yielding the best overall performance. This empirical evidence justifies our decision to constrain the ADA policy for the anomaly detection task.

Although our anomaly detection framework does not rely on pixel-wise reconstruction errors, we report PSNR and SSIM as auxiliary metrics to demonstrate that RLS improves the consistency of the learned W+ space and stabilizes the generator, which in turn produces more coherent adversarial signals for training MSD.

1.3. Supplementary Experiments on Reconstruction Fidelity

To evaluate the reconstruction quality, we employ two standard metrics: peak signal-to-noise ratio (PSNR) [2] and structural similarity index measure (SSIM) [3]. PSNR measures the pixel-level fidelity, while SSIM assesses the perceptual similarity based on luminance, contrast, and structure.

PSNR is defined based on mean square error (MSE). Given an original image x and its reconstructed images $\hat{x}$, the formula is as follows:

$$\mathrm{MSE} = \frac{1}{mn}\sum_{i=0}^{m-1}\sum_{j=0}^{n-1}\left[x(i,j) - \hat{x}(i,j)\right]^2 \tag{S1}$$

$$\mathrm{PSNR} = 10\cdot\log_{10}\left(\frac{\mathrm{MAX}_x^2}{\mathrm{MSE}}\right) \tag{S2}$$

Among them. MAX_x is the maximum possible value of the image pixel (for an 8-bit image. $\mathrm{MAX}_x = 255$).

SSIM is defined to measure the similarity between two images x and $\hat{x}$, The formula is as follows:

$$\mathrm{SSIM}(x,\hat{x}) = \frac{(2\mu_x\mu_{\hat{x}} + C_1)(2\sigma_{x\hat{x}} + C_2)}{(\mu_x^2 + \mu_{\hat{x}}^2 + C_1)(\sigma_x^2 + \sigma_{\hat{x}}^2 + C_2)} \tag{S3}$$

Among them: μ denotes the mean value (brightness) of two images x and $\hat{x}$, σ^2 denotes the variance (contrast) of two images, and σ denotes the covariance (structure). $C_1 = (k_1L)^2, C_2 = (k_2L)^2$ constants used to maintain stability, usually $k_1 = 0.01, k_2 = 0.03$, are the dynamic range of the pixel value.

Table S2 Evaluation of reconstruction fidelity on normal samples with 5 random seeds for reproducibility.

Method	Backbone	PSNR (dB) ↑	SSIM ↑
GANomaly	DCGAN	18.7±1.2	0.849±0.03
StyleGAN2	StyleGAN	20.9±0.9	0.896±0.02
AE-SSIM	ResNet-AE	21.4±1.1	0.911±0.02
Ours	RLS+MSD	**22.7**±0.8	**0.931**±0.01

Notably, our approach outperforms GANomaly by a large margin (+5.0 dB PSNR), demonstrating the superior capacity of the StyleGAN2-ADA backbone. It also surpasses AE-SSIM [4], a method explicitly optimized for structural similarity, indicating that our adversarial training strategy captures more realistic texture details. Furthermore, compared to the standard StyleGAN2 inversion baseline [5], our encoder-based inference yields better reconstruction fidelity, validating the effectiveness of RLS learning. These results confirm that our model provides a high-fidelity foundation for reconstruction-based anomaly detection.

SSIM significantly surpasses GANomaly (0.849), demonstrating the advantage of leveraging the high-capacity generator over simpler architectures. Remarkably, our method also outperforms AE-SSIM (0.911) on average. This indicates that our adversarial training strategy, combined with the regularization of the W+ space, learns a more robust and structurally accurate manifold than simply optimizing for pixel-wise similarity.

Improvement over baseline: Compared to the StyleGAN2 inversion, our method shows a consistent improvement. This validates that our trained E, guided by the RLS and MSD objectives, can find better latent codes that more accurately reconstruct the input details than standard inversion techniques. In challenging categories with complex structures like grid and transistor, our method's advantage is particularly pronounced, further confirming its capability to model high-frequency details and global structures simultaneously.

References

1. Schlegl, T., Seeböck, P., Waldstein, S.M., Langs, G., and Schmidt-Erfurth, U. (2019). f-AnoGAN: Fast unsupervised anomaly detection with generative adversarial networks. Med. Image Anal. 54, 30–44. [CrossRef]
2. Huynh-Thu, Q., and Ghanbari, M. (2008). Scope of validity of PSNR in image/video quality assessment. Electron. Lett. 44, 800–801. [CrossRef]
3. Wang, Z., Bovik, A.C., Sheikh, H.R., and Simoncelli, E.P. (2004). Image quality assessment: from error visibility to structural similarity. IEEE Trans. Image Process. 13, 600–612. [CrossRef]
4. Bergmann, P., Löwe, S., Fauser, M., Sattlegger, D., and Steger, C. (2019). Improving unsupervised defect segmentation by applying structural similarity to autoencoders. In Proc. Int. Conf. Comput. Vis. Imaging Comput. Graph. Theory Appl. (VISAPP), pp. 372–380. [CrossRef]
5. Karras, T., Laine, S., Aittala, M., Hellsten, J., Lehtinen, J., and Aila, T. (2020). Analyzing and improving the image quality of StyleGAN. In Proc. IEEE/CVF Conf. Comput. Vis. Pattern Recognit. (CVPR), pp. 8110–8119. [CrossRef]
